# “Jello^®^ Shots” and Cocktails as Ethanol Vehicles: Parametric Studies with High- and Low-Saccharin-Consuming Rats

**DOI:** 10.3390/nu5114685

**Published:** 2013-11-21

**Authors:** Nancy K. Dess, Chardonnay D. Madkins, Bree A. Geary, Clinton D. Chapman

**Affiliations:** Department of Psychology, Occidental College, 1600 Campus Road, Los Angeles, CA, 90041, USA; E-Mails: madkins@oxy.edu (C.D.M.); bree.geary@gmail.com (B.A.G.); clint@oxy.edu (C.D.C.)

**Keywords:** gelatin, ethanol, caloric compensation, flavor preference, individual differences

## Abstract

Naïve humans and rats voluntarily consume little ethanol at concentrations above ~6% due to its aversive flavor. Developing procedures that boost intake of ethanol or ethanol-paired flavors facilitates research on neural mechanisms of ethanol-associated behaviors and helps identify variables that modulate ethanol intake outside of the lab. The present study explored the impact on consumption of ethanol and ethanol-paired flavors of nutritionally significant parametric variations: ethanol vehicle (gelatin or solution, with or without polycose); ethanol concentration (4% or 10%); and feeding status (chow deprived or *ad lib*.) during flavor conditioning and flavor preference testing. Individual differences were modeled by testing rats of lines selectively bred for high (HiS) or low (LoS) saccharin intake. A previously reported preference for ethanol-paired flavors was replicated when ethanol had been drunk during conditioning. However, indifference or aversion to ethanol-paired flavors generally obtained when ethanol had been eaten in gelatin during conditioning, regardless of ethanol concentration, feeding status, or caloric value of the vehicle. Modest sex and line variations occurred. Engaging different behavioral systems when *eating* gelatin, rather than *drinking* solution, may account for these findings. Implications for parameter selection in future neurobiological research and for understanding conditions that influence ethanol intake outside of the lab are discussed.

## 1. Introduction

Naïve humans and rats voluntarily consume little ethanol at concentrations above about 6% due to its aversive flavor. Ethanol’s aversive flavor is both interesting and problematic. It is interesting because it challenges scientists to understand how and why humans and animals in diverse taxa come to consume an initially noxious substance regularly, even excessively [1,2,3]. Ethanol’s aversive flavor is methodologically problematic because it impedes observation of behaviorally and physiologically meaningful voluntary ethanol intake in the lab.

Happily, working at the methodological problem provides clues as to the substantive issues. One strategy has been selective breeding to produce rats with unusually high ethanol intake, such as UChB, P, AA, and HAD rats [4]. This strategy assures unusually high and/or low ethanol intake, facilitating the study of differential intake mechanisms and sequelae. Another strategy has been the development of parameters and procedures that effectively increase ethanol intake. For instance, using low-concentration or sweetened ethanol vehicles [5,6,7] boosts ethanol intake and models initiation of ethanol consumption via vehicles that mask ethanol’s noxious flavor, including beer, wine, cocktails, or carbonated “alcopops” that are drunk and fermented or “crocked” fruit or “jello shots” that are eaten. A third strategy involves pairing an arbitrary flavor with ethanol (flavor conditioning) and then examining the expression of preference (or aversion) toward the ethanol-paired flavor [8,9,10,11,12]. Methodologically, this strategy removes ethanol’s flavor as a motivational factor during testing, simplifying interpretation of test behavior *vis à vis* flavor-taste or flavor-nutrient associations, metabolic status, or pharmacological effects [13,14]. Substantively, flavor conditioning models the modification of preferences and motivations through experience with ethanol-containing beverages or foods.

The present study blended all three strategies to generate data of value to researchers seeking to “tune” procedures for enhancing motivation to ingest ethanol. Rats differentially predisposed to consume ethanol were studied. Occidental high- and low-saccharin-consuming rats (respectively, HiS and LoS), which have been selectively outbred for over 40 generations on a voluntary saccharin intake phenotype [15], differ in voluntary ethanol intake (HiS > LoS) [16]. These lines comprise a convenient model for studying ethanol intake and its association with responses to other sweet or bittersweet substances, from gustatory evaluation to withdrawal [3,17,18,19,20,21]. We also have tested these lines in an ethanol-paired flavor conditioning procedure consisting of overnight exposures to two fruit-flavored solutions, one with ethanol and one without ethanol, each presented five times in strict alternation [17,21]. In a subsequent two-bottle test (both flavors in aqueous solution), LoS rats’ behavior toward an ethanol-paired flavor varies from preference (preference score > 0.50) to indifference or aversion (preference score ≤ 0.50) depending on ethanol concentration, the relative novelty of the flavors, and feeding status. For instance, LoS males express a preference for a flavor paired with 10% ethanol only if exposure to the flavors is matched (“yoked”) during conditioning such that the flavors are equally familiar. In addition, LoS rats express a *preference* for an ethanol-paired flavor when freely fed but express an *aversion* to it if tested during refeeding after acute chow deprivation. By comparison, HiS rats’ preference for an ethanol-paired flavor is more consistent across conditioning and testing procedures. These findings comport with other evidence that LoS rats are more responsive than HiS rats to perturbations of metabolic status [22,23,24]. They also show that an *association*—rather than preference or aversion—between ethanol and an arbitrary flavor is learned during conditioning, and its behavioral expression in a later test can vary dramatically depending on intervening events and test conditions.

In a previously unpublished preference test, 4% ethanol-paired or unpaired flavors were presented in 10% ethanol (rather than water) to freely feeding rats. Both lines consumed more ethanol in the ethanol-paired flavor vehicle than in the equally familiar control flavor (for ethanol-paired flavor as a proportion of total intake, HiS, 0.78 ± 0.04; LoS, 0.75 ± 0.03). This finding demonstrates that preference for a 4% ethanol-paired flavor can promote consumption of a higher, unconditionally aversive ethanol solution. Other research has shown that a cue associated with ethanol promotes instrumental performance to obtain ethanol [25]. Flavor does not “explain” humans’ alcohol intake, but it contributes to it, especially during early stages of alcohol consumption [26,27,28,29,30]. This flavor conditioning preparation, then, allows assessment of ethanol-related motivation with a proxy taste—eliminating complexities of ethanol’s concentration-dependent flavor—and models the role in alcohol intake of dispositional differences and of learning during initial alcohol experiences.

In our prior experiments, conditioning and test vehicles were noncaloric aqueous solutions. The present study builds on those findings by examining ethanol intake and ethanol-paired flavor preference as nutritionally significant parameters are varied: eating (in gelatin) *versus* drinking (in solution) ethanol and test flavors; ethanol concentration (4% or 10%); feeding status (*ad lib*. feeding or chow deprivation) during conditioning and/or preference tests; and calorie-rich (10% polycose) *versus* minimally caloric ethanol vehicles. The pattern of results speaks to the importance (or lack thereof) of taste, calories, the behavioral/motivational system engaged, and dispositional differences in proclivity to consume ethanol.

## 2. Experiment 1

This series began with an experiment conducted to determine whether our previous findings for flavors paired with ethanol in aqueous solution would extend to flavors paired with ethanol in a semi-solid food—a gelatin matrix. In a laboratory setting, people and rats readily consume physiologically significant amounts of ethanol in semi-solid gelatin. In research to date, the gelatin’s palatability and calories come from sucrose for humans and from the polysaccharide polycose for rats [31,32,33]. Outside of the lab, “jello shots” are a popular way of consuming alcohol among youth [26]. To our knowledge, how eating ethanol in fruity, calorie-laden gelatin affects later flavor-based choice behavior has not been experimentally examined. Doing so will bear on whether conditioned flavor preferences might contribute to increased alcohol use by youth consuming “jello shots” and, if so, whether they do so differentially as a function of dispositional propensity to consume alcohol.

We also examined compensation for calories consumed as ethanol/polycose gelatin by HiS and LoS rats. When Rowland *et al.* [32] gave rats 24 h access to polycose gelatin containing 5% or 10% ethanol, both male and female rats compensated for calories consumed as gelatin through graded reductions in chow intake; among males (but not females), total caloric intake was actually below baseline chow caloric intake when 10% polycose/10% ethanol gelatin was available (overcompensation). Rats’ sensitivity to the gelatin as a source of calories demonstrates ethanol’s unique status among psychoactive substances as food as well as drug. We used Rowland *et al.*’s recipe for 10% polycose gelatin with and without 10% ethanol for direct comparability to their results. On the basis of prior work with HiS and LoS rats, we predicted that LoS rats would show better compensation than HiS rats for calories consumed as gelatin.

### 2.1. Method

#### 2.1.1. Rats

In this and each of the following experiments, adult (60–90 days of age) rats from Generations 42 and later in our colony were used; littermates (5–11 litters per line) were balanced across experimental conditions. They were individually housed in stainless steel cages on a 12:12 light/dark cycle (0700–1900 light). Daily water and baseline bodyweight were measured, yielding just two significant line differences in water intake (in opposite directions) and one in bodyweight in 14 experiments; therefore, no detailed information on those data is provided (available on request). All procedures complied with a protocol approved by the Occidental College Institutional Animal Care and Use Committee.

Adult female HiS (*n* = 11) and LoS (*n* = 13) rats were used in Experiment 1. Baseline chow intake was measured in addition to water and bodyweight.

#### 2.1.2. Materials

Gelatin was prepared by dissolving Knox^®^ unflavored gelatine (3% wt/wt; Kraft Food Group, Northfield, IL, USA) in boiling water, cooling with tap water, and adding unsweetened cherry or grape KoolAid (0.25% wt/wt; Kraft Food Group, Northfield IL, USA). In this and the following experiments except where noted otherwise, Polycose^®^ (10% wt/wt; Abbott Laboratories, Abbott Park, IL, USA) also was added to the gelatin mixture. Ethanol gelatin contained 10% wt/wt ethanol. Gelatin was poured into glass jars (~100 g), which were capped and refrigerated until the gelatin was set. Jars were secured in cages with metal holders. Pelleted chow (Purina 5001 Rodent Chow; Purina Mills, St. Louis MO, USA) and tap water were continuously available. Caloric densities were 0.49 kcal/g for no-ethanol/polycose gelatin, 1.03 kcal/g for ethanol/polycose gelatin, and 3.36 kcal/g for chow.

#### 2.1.3. Procedure

From late afternoon to early morning (approximately 1600 to 800) on each of the next 10 days, rats had access to a glass jar containing fruit-flavored gelatin. Cherry and grape gelatin were presented five times each, and for each rat, one of these flavored gelatins contained ethanol. Ethanol gelatin and no-ethanol gelatin were presented in strict alternation (one on odd days, one on even days), with ethanol-paired flavor (cherry or grape) and type of gelatin provided on the first day balanced across littermates. Gelatin and chow intake were measured daily.

After the last conditioning trial, rats were given two days of free access to only chow and water before a 24-h two-jar flavor preference test. Chow, water, and two jars containing, respectively, cherry and grape flavored 10% polycose gelatin (with left/right positions balanced) were available during the test. Gelatin intake was measured. We did not correct for evaporation [32], which we estimate to be ~1.5 g per 18–24 h, so gelatin and ethanol consumption means reported in this paper are slight overestimates; however, our conclusions concern ordinal differences and as such stand without adjusting for evaporation. Moreover, given the specific gravity of ethanol, weight/weight gelatin formulations used here represent slightly higher ethanol concentrations than were used in prior work with 4% and 10% volume/volume concentrations, offsetting small differences owing to evaporation.

### 2.2. Results and Discussion

Data were evaluated for significance at α = 0.05. Test statistics with *P* ≤ 0.05 are reported in the text. *P* was evaluated for significance after Greenhouse-Geisser adjustment to *df*s for repeated measures effects with *df* > 1, and after Bonferroni adjustment for multiple contrasts to interpret interactions and main effects with *df* > 1. As observed previously [17,21], daily caloric intake was higher among LoS rats (77.9 ± 3.1 kcal) than HiS rats (67.7 ± 2.8 kcal), *t*(22) = 2.37. This result points to lower metabolic efficiency among LoS rats, the mechanisms of which remain to be determined.

**Figure 1:**
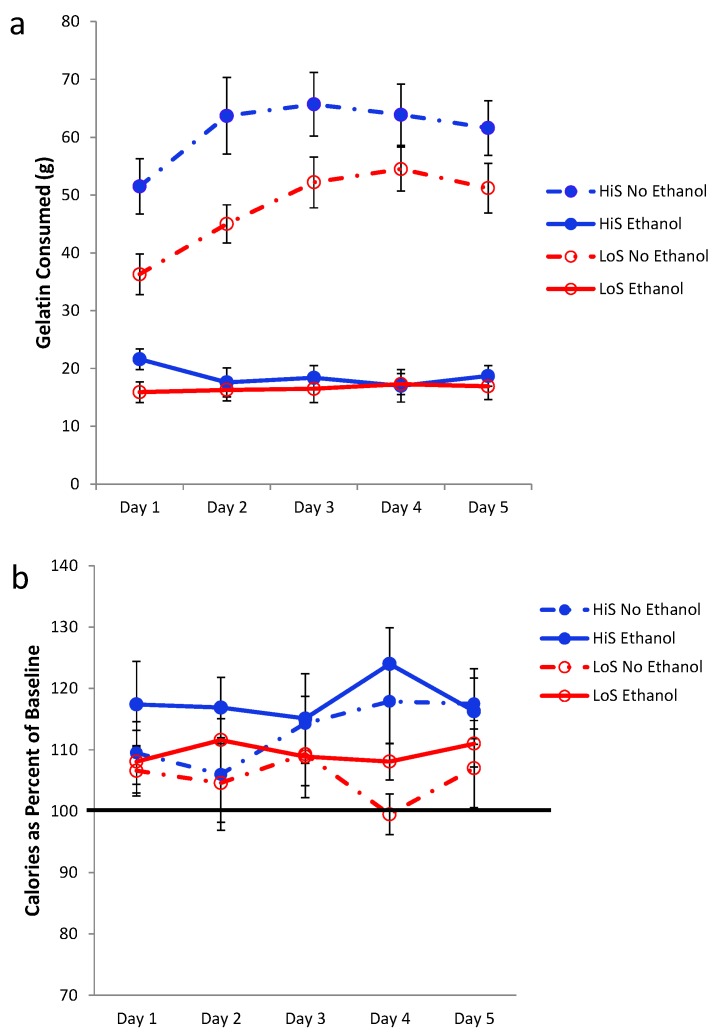
Intake of 10% ethanol/polycose *versus* no-ethanol/polycose gelatin (Panel **a**) and total calorie intake (Panel **b**). Day 2 means are shown, but those data were omitted from the ANOVA because some rats’ chow intake was inadvertently not recorded.

Rats readily consumed both kinds of gelatin but consumed more no-ethanol than ethanol gelatin (intake in grams, Figure 1a). This difference grew over days as no-ethanol gelatin intake increased while ethanol gelatin intake stayed the same. Even allowing for the lower caloric density of no-ethanol gelatin (roughly half of the ethanol gelatin), more calories were consumed as no-ethanol gelatin. HiS rats consumed more no-ethanol gelatin than did LoS rats. 

A mixed-design analysis of variance (ANOVA) with line, gelatin type, and day as variables yielded main effects of line and type and a line × type interaction, *F*s(1,22) = 4.33, 285.82, 6.77, respectively; contrasts showed that the no-ethanol > ethanol difference was significant for both lines. The day main effect and type × day interaction also were significant, *F*s(4,88) = 6.36 and 11.88, respectively; the no-ethanol > ethanol difference was significant on all trials. Average daily ethanol intake expressed as dose (g/kg) was 6.4 ± 0.6 for HiS rats and 5.8 ± 0.7 for LoS rats; these values are comparable to the ethanol doses consumed in aqueous solution in our original ethanol-paired flavor conditioning study [21].

Total calorie intake (gelatin + chow), expressed as a percent of baseline calorie intake (chow only), is shown in Figure 1b. Although rats ate more no-ethanol gelatin than ethanol gelatin (Figure 1a), they consumed more total calories when ethanol gelatin was available. LoS rats compensated for no-ethanol gelatin intake by reducing chow intake more so than did HiS rats. Following a strategy we have used previously [17], these data were subjected to two analyses, the first of which compares groups/conditions to each other and the second of which compares each group/condition to a meaningful benchmark value. First, a mixed design analysis of variance (ANOVA) was used to evaluate line, gelatin type, and day effects. This ANOVA yielded a main effect of gelatin type, *F*(1,22) = 5.95. This effect was stable over days. Second, a one-sample *t* test was used to compare the overall mean for each gelatin type in each line to 100%. These tests allow identification of conditions in which HiS and/or LoS rats compensated (=100%), undercompensated (>100%), or overcompensated (<100%) for access to gelatin with changes in chow intake. HiS rats’ calorie intake exceeded baseline when either ethanol or no-ethanol gelatin was available, *t*s(10) = 4.30 and 3.36, respectively. LoS rats’ calorie intake also exceeded 100% when ethanol gelatin was available, *t*(12) = 2.41, but not when no-ethanol gelatin was available. Analysis of the first gelatin day (whether ethanol or no-ethanol) showed “overeating” by both lines [mean *versus* 100% for HiS, *t*(10) = 2.85; LoS, *t*(12) = 2.48], suggesting that LoS rats’ compensation for no-ethanol gelatin intake improved over days even as gelatin intake rose. Still, overall, compensation was inferior to what Rowland *et al.* [32] observed.

Here and below, ethanol-paired flavor preference in the two-jar test was assessed in the same two-pronged manner as was caloric compensation, *i.e.*, group means were compared (with an independent *t* test or ANOVA, as appropriate) and then each group’s mean was compared to indifference (preference score of 0.5) with a one-sample *t* test. HiS and LoS lines did not differ from each other, and neither preferred the ethanol-paired flavor to the no-ethanol flavor. LoS rats were indifferent [0.45 ± 0.06], and HiS rats had a marginally significant *aversion* to the ethanol paired flavor [0.37 ± 0.07, *t*(10) = 1.85, *P* = 0.09]. These results violated our expectation of an ethanol-paired flavor preference, as previously obtained in both lines—most reliably in HiS rats—using cherry or grape-flavored ethanol solutions during conditioning, with chow freely available [17,21].

The ensuing experiments were designed to identify which difference(s) between prior studies using solutions and this gelatin preparation account for the indifference, leaning toward aversion, observed here. Two series of experiments were conducted. The first series employed the same basic flavor conditioning paradigm as in Experiment 1, in search of parameters that would yield preference for an ethanol-paired flavor. The second series examined relative intake of gelatins and solutions as a function of ethanol and/or polycose content, as a means of exploring the role of unconditioned flavor effects (acceptability, palatability) in preference/aversion expressed in the conditioning paradigm.

## 3. Experiments 2–8: Flavor Conditioning Series

One possible explanation for the absence of an ethanol-paired flavor preference is the unconditionally aversive qualities of 10% ethanol. A problem with this explanation is that HiS and LoS rats conditioned with 10% ethanol in solution express an ethanol-paired flavor preference despite the noxious taste of the conditioning solution [17,21]. Potentially, though, a stronger aversive taste-taste association develops in a gelatin medium, preventing expression of a flavor preference. A somewhat more likely explanation centers on differential intake of the 10% ethanol and no-ethanol gelatins during conditioning. Examining caloric compensation in Experiment 1 required providing unlimited access to both gelatins during conditioning. Rats consumed fewer calories as ethanol gelatin than no-ethanol gelatin, and lower caloric intake associated with the former could contribute to preference for the alternative flavor [34]. In addition, the relative novelty of the ethanol-paired flavor could have reduced selection of it in the choice test (neophobia). Indeed, using solutions during conditioning, preference for an ethanol-paired flavor was less reliable when it was relatively novel [21]. In that study, only LoS rats displayed sensitivity to relative novelty. However, in Experiment 1, the ethanol/no-ethanol gelatin difference was greater among HiS, so it is at least possible that the larger difference in relative novelty contributed to their tendency to choose the no-ethanol flavor in the test. In Experiments 2–8, a yoking procedure was used to roughly equate each rat’s exposure to the two flavors prior to the preference test, which also ensured that both greater caloric density and total calorie intake would be associated with the ethanol-paired flavor.

Table 1 summarizes Experiment 1 and subsequent manipulation of variables that might influence choice of an ethanol-paired flavor when the conditioning medium is gelatin. Baseline measurement of water intake and bodyweight, the 10-day alternating flavor conditioning procedure, days off, and two-jar flavor preference tests were as described for Experiment 1. In Experiments 2–8, more than one flavor preference test was administered, with one day of free access to chow and water separating the tests; when two gelatin tests were given with different conditions (e.g., *ad lib*. feeding *versus* chow deprivation), test order was counterbalanced. Given the uniformity of basic conditioning and test procedures, only key differences from preceding experiments are explained here. In the 10 conditioning experiments conducted, three HiS rats (in different experiments) and two LoS rats (in different experiments) ate virtually none of the ethanol gelatin and were excluded from data analyses.

### 3.1. Experiment 2

Experiment 2 directly replicated Experiment 1 with two exceptions. First, after the two-gelatin flavor test and a day off, rats received a two-fluid flavor test (0.25% cherry and grape KoolAid in tap water). Second, during conditioning, no-ethanol gelatin intake was yoked to ethanol gelatin intake. Female HiS and LoS rats (*n*s = 15) weighing approximately 305 g (no line difference) were given a full jar of ethanol gelatin on Day 1; on Day 2, each rat was given a ration of no-ethanol gelatin equal to the amount of ethanol gelatin (in grams) she had consumed the day before. Yoking was repeated for each of the remaining four ethanol/no-ethanol pairs of conditioning days. Because the caloric density of the ethanol gelatin was higher than that of the no-ethanol gelatin, yoking meant that unlike Experiment 1, rats consumed more calories as ethanol gelatin than no-ethanol gelatin. Greater caloric density and total calorie intake should favor a preference for an associated flavor [8,13,34,35]. If, in Experiment 1, either having consumed more calories as no-ethanol gelatin or the greater familiarity of the no-ethanol gelatin flavor contributed to indifference or aversion to the ethanol-paired flavor, yoking should increase ethanol-paired flavor preference—at minimum, it should eliminate rejection of it in favor of the no-ethanol flavor.

**Figure 2:**
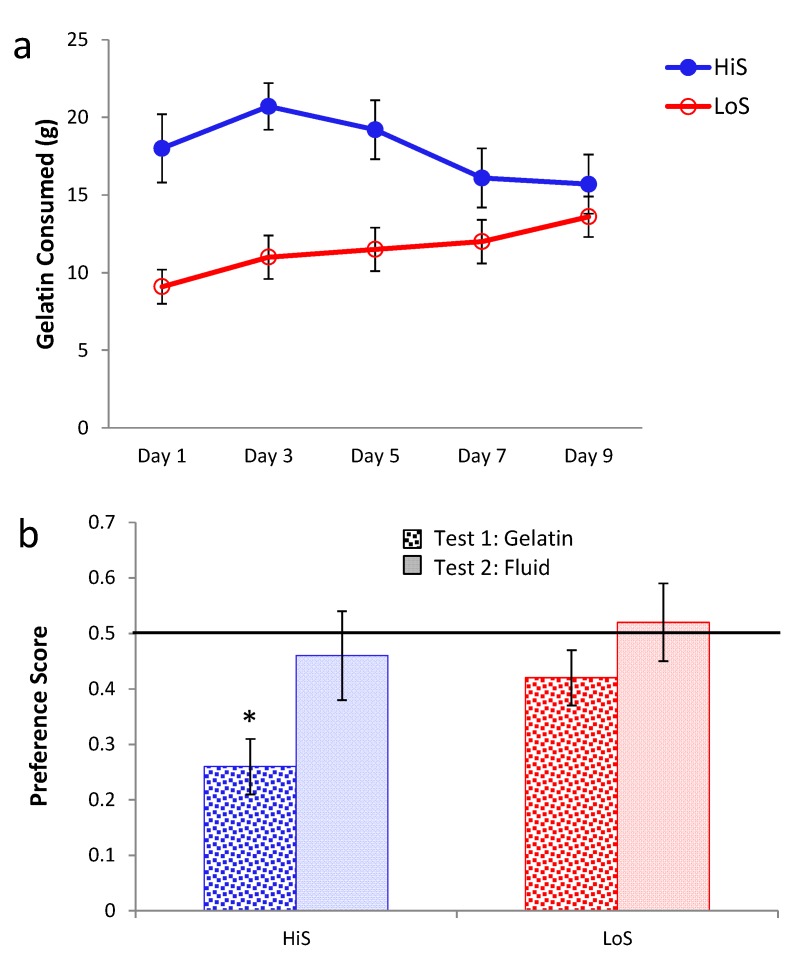
Intake of 10% ethanol/polycose gelatin during conditioning (Panel **a**) and ethanol-paired flavor preference in polycose gelatin and no-polycose fluid (Panel **b**) in Experiment 2. Mean preference scores differing significantly from indifference (0.5) are marked with an asterisk (*).

Ethanol gelatin intake during conditioning is shown in Figure 2a. HiS rats consumed more gelatin than did LoS rats [line main effect, *F*(1,22) = 11.32]. However, the line × day interaction also was significant, *F*(4,88) = 5.55: Bonferroni-adjusted contrasts showed that LoS rats’ intake did not change significantly across Days 1–5, whereas for HiS rats, intake on Day 2 was significantly higher than on Days 4 and 5. Ethanol-paired flavor preference is shown in Figure 2b. HiS rats rejected the ethanol-paired flavor in favor of the no-ethanol paired flavor; in the subsequent two-solution test, they were indifferent, a result to which extinction during the first test might have contributed. LoS rats were indifferent in both tests. A line × test medium ANOVA yielded a main effect of test medium, *F*(1,22) = 10.00. One-sample *t* tests comparing each of the four means to 0.5 showed indifference except for significant aversion among HiS rats tested with gelatin, *t*(14) = 4.99.

**Table 1:** Summary of Flavor Conditioning and Unconditioned Flavor Test series. Procedural changes within the first series are **bolded**. The gelatin type given to rats as a ration (yoked to *ad lib*. intake of the alternative on the preceding day) is shown in 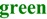. Preferences/increases are shown in 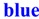, aversions/decreases in 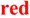.

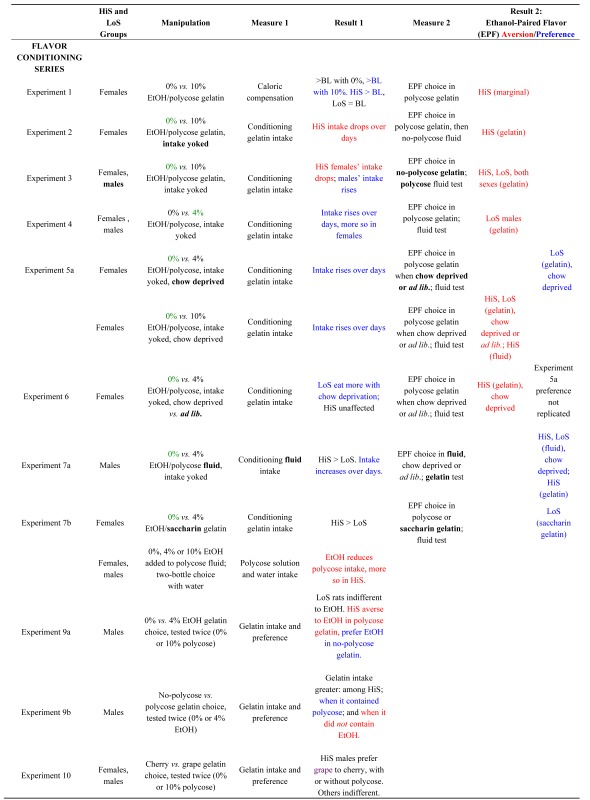

Thus, with flavor exposure during conditioning matched and total flavor-associated calorie intake reversed from Experiment 1, HiS rats’ tendency toward aversion to the ethanol-paired flavor—which also signaled higher caloric density—was strengthened, not weakened. This aversion is surprising, even if eating the 10% ethanol gelatin shifts brain ethanol levels toward those resulting from drinking 4% solution [31], because 4% and 10% ethanol *solutions* both support a preference in both lines [21].

### 3.2. Experiment 3

Experiment 3 directly replicated Experiment 2 with two exceptions. First, male as well as female rats were used (*n*s = 13–14). Second, test gelatins were made without polycose and test fluids were made with polycose. The polycose gelatin used in Experiments 1 and 2 is a palatable, caloric flavor medium. In contrast, the flavored test solutions used in our previous studies [17,21] were neither palatable nor caloric. Perhaps, then, rats only express a preference for an ethanol-paired flavor when the test medium otherwise has little positive incentive value. If so, rats might express an ethanol-paired flavor preference when tested with minimally caloric no-polycose gelatins and an aversion when tested with polycose fluids.

**Figure 3:**
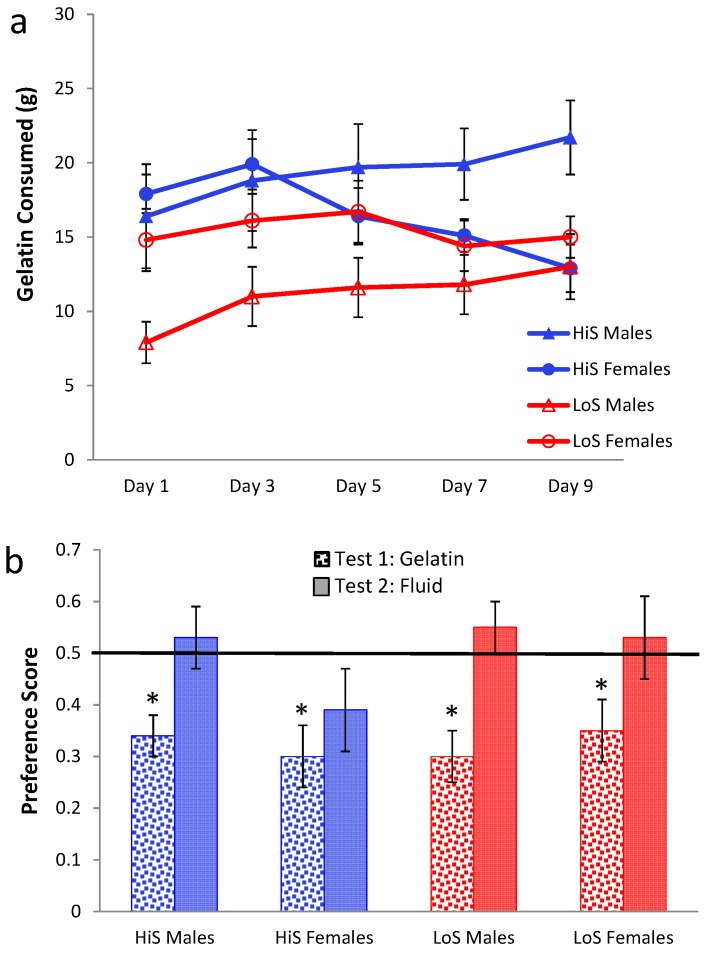
Intake of 10% ethanol/polycose gelatin during conditioning (Panel **a**) and ethanol-paired flavor preference in no-polycose gelatin and polycose fluid (Panel **b**) in Experiment 3.

Ethanol gelatin intake during the yoked conditioning procedure is shown in Figure 3a. As in Experiment 2, female rats’ gelatin intake decreased, somewhat more so among HiS. In contrast, males’ intake increased, with HiS males eating more than LoS males throughout. A line × sex × day ANOVA yielded a line main effect, *F*(1,50) = 6.79, and interactions of sex with line, *F*(1,50) = 4.01, and day, *F*(4,200) = 5.77. The interactions were interpreted with Bonferroni-adjusted contrasts. HiS males’ overall gelatin intake exceeded LoS males’; marginal means for females did not differ. Females’ intake decreased from Day 2 to Day 5; although the interaction with line was not significant, this decrease clearly derives more from HiS than from LoS females, whose intake was relatively stable. Males’ intake increased significantly from Day 1 to Day 5. In contrast, increasing intake of ethanol gelatin among males is consistent with the development of a preference for the ethanol-paired flavor. Yet, as shown in Figure 3b, males and females in both lines rejected the ethanol-paired flavor in favor of the no-ethanol flavor in the no-polycose gelatin choice test. A line × sex × test medium ANOVA yielded a main effect of test medium, *F*(1,50) = 22.13. Comparison of each mean to 0.5 confirmed aversion when all four groups were tested with gelatin, *t*s(14) ≥ 2.69, and indifference when tested with solution. Testing with no-polycose gelatin produced more uniform aversion to the ethanol-paired flavor rather than “unmasking” a preference for it, and testing with polycose fluids still yielded indifference.

### 3.3. Experiment 4

Experiment 4 directly replicated Experiment 3 with three exceptions. First, gelatin was made with 4% ethanol (0.9 kcal/g). Unlike 10% ethanol solution, 4% ethanol solution is unconditionally preferred to water by both HiS and LoS rats [16]; like 10% ethanol, it supports expression of ethanol-paired flavor preference among freely chow fed rats [21]. Second, during conditioning, yoking was reversed such that no-ethanol gelatin was provided on odd days and a matched ration of 4% ethanol gelatin was provided on even days. This change was made because rats’ preference for 4% ethanol over water leads reasonably to the expectation that they would consume more 4% gelatin than no-ethanol gelatin. Finally, we returned to testing with polycose gelatins and no-polycose fluids. If a relatively unpalatable conditioning and/or test gelatin limits expression of an ethanol-paired flavor preference, then using more palatable media might reveal a preference. We know that rats will express an ethanol-paired flavor preference in a no-polycose fluid test [17,21] and so returned to that type of fluid test as well.

**Figure 4:**
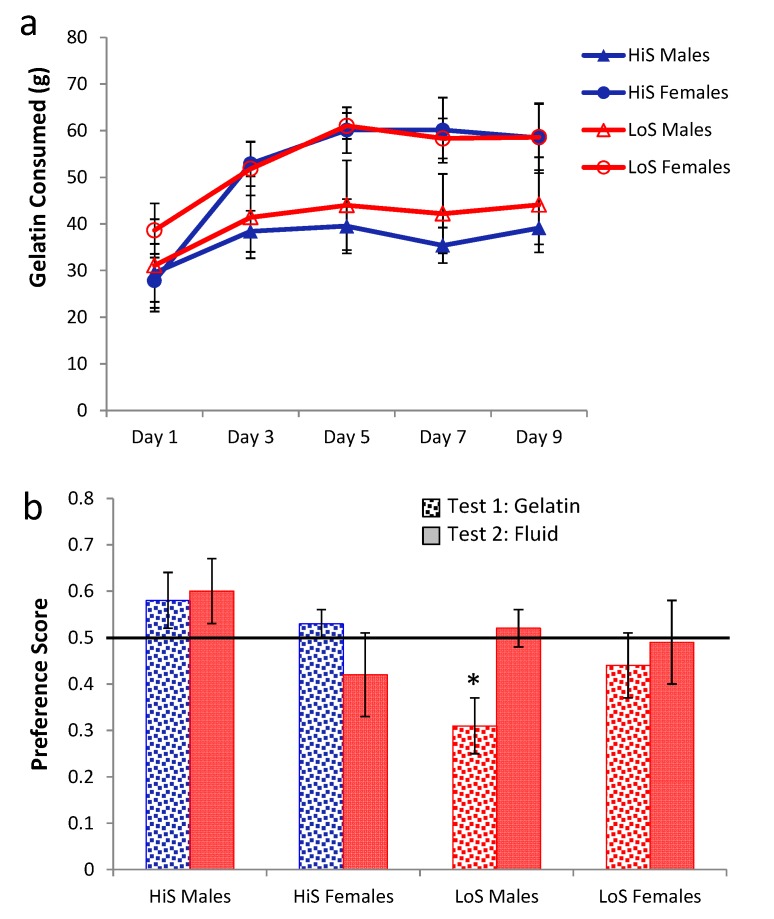
Intake of 4% ethanol/polycose gelatin during conditioning (Panel **a**) and ethanol-paired flavor preference in polycose gelatin and no-polycose fluid (Panel **b**) in Experiment 4.

No-ethanol gelatin intake during conditioning by HiS and LoS females and males (*n*s = 7 or 8) is shown in Figure 4a. Overall intake was 2–3 times greater than for 10% ethanol gelatin in Experiments 1–3 and was greater among females than males. Intake increased across days in both males and females, somewhat more so in the latter. Intake was comparable in HiS and LoS rats.

A line × sex × day ANOVA yielded main effects of sex, *F*(1,27) = 5.82, and day, *F*(4,108) = 19.90, and a sex × day interaction, *F*(4,108) = 3.90. The interaction was ordinal; Bonferroni-adjusted contrasts showed that in both males and females, intake was significantly lower on Day 1 than on Day 2, which was not significantly different from subsequent days. Expressed as ethanol dose, by the end of conditioning females were consuming a slightly higher dose than they had of 10% ethanol in Experiment 1 (7.6 ± 0.6 g/kg) and more than males (3.6 ± 0.5). This sex difference and the male’s ethanol dose are comparable to results in our original ethanol-paired flavor conditioning study [21].

Ethanol-paired flavor preference is shown in Figure 4b. Results for LoS males were the same as when 10% ethanol gelatin was used during conditioning: They were averse to the ethanol-paired flavor in gelatin and were indifferent to it in the subsequent fluid test. All other groups were indifferent in both tests. A line × sex × test medium ANOVA yielded a line × test medium interaction, *F*(1,27) = 5.66, and Bonferroni-adjusted contrasts showed that the lines differed in the gelatin test (HiS > LoS) but not in the fluid test. Comparison of each mean to 0.5 confirmed significant aversion when LoS males were tested with gelatin, *t*(7) = 3.07, and indifference in all other cases. An unforeseen occurrence during conditioning, however, leaves these test results open to interpretation: Whereas in Experiments 2 and 3 nearly all rats consumed all of their no-ethanol ration on even-numbered days, in Experiment 4 most rats left most of the 4% ethanol rations uneaten. Contrary to the high acceptability of 4% ethanol solution, 4% ethanol did not seem to increase the acceptability of polycose gelatin. The Unconditioned Flavor Test Series (Experiments 8–10) below addresses unconditioned flavor acceptability and preference issues further. Here, the point is that despite the attempt at yoking, the ethanol-paired flavor was somewhat less familiar than the no-ethanol flavor, which would tend to reduce preference for it, especially among LoS rats [21]. Thus, neophobia could account for LoS males’ rejection of the ethanol-paired flavor, and failure of other groups to prefer it.

### 3.4. Experiment 5

Experiments 5a and 5b directly replicated Experiment 4 with the following exceptions. First, only females were used (*n*s = 15–19). Second, yoking was reversed (ethanol gelatin on odd days, no-ethanol rations on even days). Third, rats were chow deprived during gelatin access (free access to chow otherwise). In our studies with flavored ethanol solutions, those solutions were the only source of fluid available overnight and, because intake was lower than baseline water intake, chow intake likely was lower than baseline. In contrast, in Experiments 1–4, gelatin was not the only source of calories; chow and water were available, and total caloric intake was at or above baseline levels (Experiment 1). If consuming an ethanol-paired flavor during mild caloric restriction contributed to a preference for it in our prior work [8,10,12,35], then removing chow during gelatin intake might yield a preference. Fourth, the design was run with both 4% ethanol (Experiment 5a) and 10% ethanol (Experiment 5b). Finally, flavored gelatin preference tests were conducted twice, during free access to chow and chow deprivation, with order balanced, prior to the fluid test. Negative energy balance can enhance expression of preferences for an ethanol-paired flavor [8,10,11], so testing during chow deprivation might reveal a preference based on gelatin conditioning that is not expressed when rats are freely feeding.

**Figure 5:**
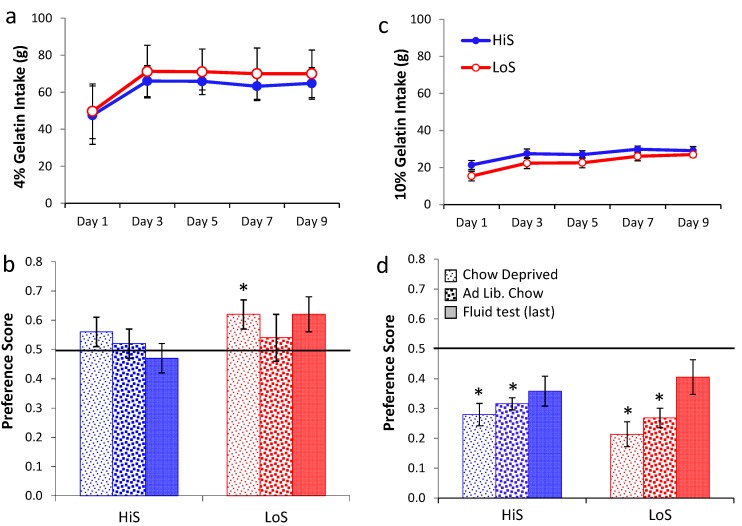
Ethanol/polycose gelatin intake by chow-deprived rats in Experiments 5a (4% EtOH) and 5b (10% EtOH) (respectively, Panel **a** and **c**; legend in latter). Ethanol-paired flavor preference in polycose gelatin as a function of feeding status, and in a final no-polycose fluid test in Experiments 5a and 5b (respectively, Panel **b** and **d**; legend in latter).

Ethanol gelatin intake during conditioning is shown Figure 5a (4%) and 5c (10%). At both concentrations, ethanol gelatin increased over days. Line × day ANOVAs yielded a significant main effect of day at both 4%, *F*(4,128) = 29.35, and 10%, *F*(4,132) = 10.70. No other effects were significant. Preference for a flavor paired with 4% ethanol is shown in Figure 5b. The first significant ethanol-paired flavor preference in this study was observed here, when LoS rats were conditioned and tested chow deprived. Otherwise, indifference prevailed. A line × test condition (gelatin chow deprived, gelatin *ad lib*., fluid) ANOVA yielded no significant effects. Comparison of each mean to 0.5 revealed a significant preference for LoS/Chow Deprived, *t*(14) = 2.22.

Rats conditioned with 10% ethanol were averse to the ethanol-paired flavor in gelatin regardless of feeding status, with stronger aversion expressed when rats were tested chow deprived. Trends toward aversion also occurred during the fluid test but neither achieved statistical significance (*P*s > 0.058). Because some rats in Experiment 5b were inadvertently tested with fluid containing polycose, a line × feeding status ANOVA was run on data from the gelatin tests, and an independent *t* test was used to compare the subset of LoS and HiS for which valid fluid test data were available. The ANOVA yielded a significant feeding status effect, *F*(1,33) = 4.54; no other differences were significant. Comparison of means to 0.5 confirmed aversion in both lines during both gelatin tests, *t*s > 4.

Improved yoking (compared to Exp. 4) did not unmask preference for a flavor paired with 4% ethanol among HiS rats. Nor did chow deprivation during gelatin testing unmask preference for a flavor paired with 10% ethanol; in fact, it strengthened choice of the no-ethanol flavor. These results do suggest constraints within which preference for an ethanol-paired gelatin flavor might occur—specifically, when conditioning gelatin contains a low ethanol concentration (4%) and when rats sensitive to feeding status (LoS) are conditioned and tested during caloric deficit.

### 3.5. Experiment 6

**Figure 6:**
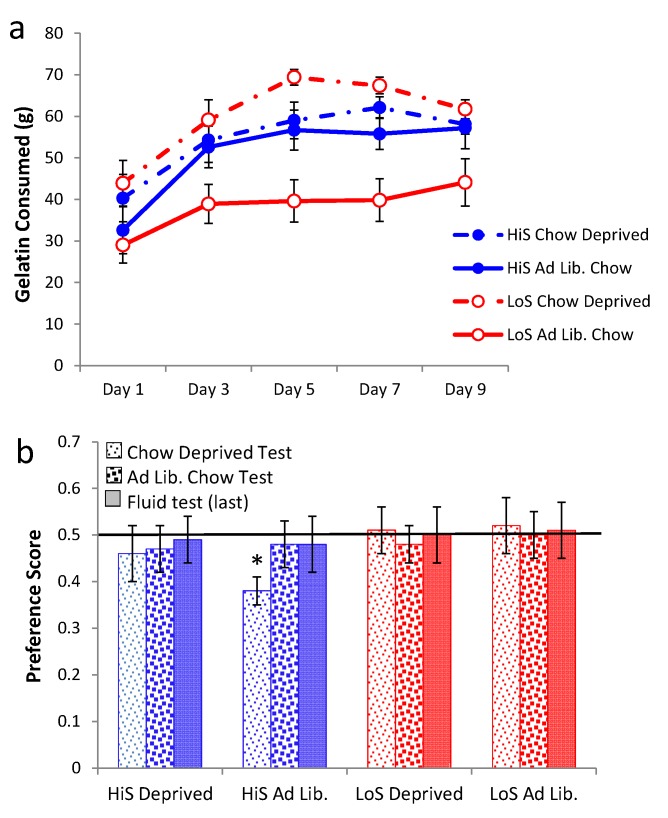
Intake of 4% gelatin during conditioning (Panel **a**) and ethanol-paired flavor preference in gelatin and fluid (Panel **b**) in Experiment 6.

Experiment 6 was designed to determine whether the ethanol-paired flavor preference observed within those constraints in Experiment 5a is properly attributed to conditioning with chow deprivation, by directly comparing rats conditioned with chow deprivation (as in Experiment 5a) to rats conditioned with continuous access to chow (as in Experiment 4). Procedures were the same as in Experiment 5a except for experimental manipulation of feeding status: HiS and LoS female rats (*n*s = 14) were assigned to either *ad lib*. chow or chow deprivation during conditioning, with litters balanced across the two conditions. Intake of 4% ethanol gelatin during conditioning is shown in Figure 6a. Gelatin intake increased over days, and chow deprivation increased gelatin intake among LoS rats. Interestingly, chow deprivation did not affect gelatin intake among HiS rats, another indication of their reduced behavioral regulation of energy balance (Experiment 1) [21,22,23]. A line × feeding status × day ANOVA yielded main effects of feeding status, *F*(1,61) = 11.44, and day, *F*(4,244) = 39.43, and a line × feeding status interaction, *F*(1,61) = 5.72.

Ethanol-paired flavor preference is shown in Figure 6b. Among rats fed *ad lib*. during conditioning, LoS rats were indifferent, and HiS rats expressed frank aversion when chow deprived during gelatin testing. Rats that were chow deprived during conditioning were indifferent in all test conditions; the preference observed among chow-deprived LoS rats in Experiment 5 was not replicated. A line × test condition ANOVA yielded no significant effects. Comparison of each mean to 0.5 yielded a significant difference for HiS/*Ad Lib*. Conditioned/Chow Deprived Test, *t*(15) = 4.15. No other means differed from 0.5.

### 3.6. Experiment 7

A viable explanation for failure to observe a reliable ethanol-paired flavor preference after gelatin conditioning is that having 10% polycose in both ethanol and no-ethanol gelatin nullifies whatever differences favor later choice of a flavor associated with ethanol. For instance, flavors can be weaker in gelatin than in solution, at least in humans [36]. The preceding experiments yielded many deviations from indifference, predominantly in the direction of aversion—especially, surprisingly, among HiS rats. Apparently, having polycose in the gelatins does not simply make their flavors undetectable or indiscriminable. Rather, by increasing caloric density and/or the palatability of the medium, a polycose base might increase the salience of ethanol-associated factors—noxious sensory qualities, for instance—that support aversion and/or attenuate preference. This account is consistent with our having observed ethanol-paired flavor preference among freely feeding HiS and LoS rats when noncaloric, relatively unpalatable solutions were used during conditioning [17,21]. The absence of polycose rather than the fluid medium in those studies might account for the discrepancy between those and the present findings.

Experiment 7 examined flavor preference after conditioning with polycose solutions (Experiment 7a) and with no-polycose gelatins (Experiment 7b). Rats in Experiment 3 consumed substantial amounts of no-polycose test gelatins (overall average ~26 g); however, those rats had a history of eating polycose gelatin, and pilot testing showed that many naive rats consume trivial amounts of KoolAid-flavored no-polycose gelatin. Thus, to achieve intake sufficient to support flavor learning, saccharin was added to the no-polycose gelatins in Experiment 7b. We expected HiS rats to consume more gelatin than did LoS rats given selection on a saccharin phenotype, but because HiS rats consumed more polycose gelatin than did LoS rats in the preceding experiments, such a difference would facilitate comparison of these results with earlier ones. Moreover, to date, we have not compared HiS and LoS rats on consumption of a saccharin flavored food that was eaten rather than drunk, so Experiment 7b bore on the generality of their differential propensity to consume saccharin-flavored substances.

In Experiment 7a, the same yoked-intake design was used as in Experiments 2–6, substituting 10% polycose (wt/vol) solutions for polycose gelatins: Male rats (*ns* = 12) received 4% ethanol/polycose solution flavored with cherry or grape KoolAid (0.25% wt/vol) on each odd day and an equal-weight ration of the alternate flavor in no-ethanol/polycose solution the next day. Two preference tests with flavored no-polycose fluids were conducted, once during chow deprivation and once during *ad lib*. feeding, with order balanced; a final preference test with polycose gelatin then occurred. Conditioning in Experiment 7b was identical to Experiment 4 except that only females (*n*s = 11 or 13) were used and gelatins were made with 0.2% (wt/wt) sodium saccharin (Sigma Aldrich Inc., St Louis, MO, USA) instead of polycose. Flavor preference tests were conducted with polycose gelatins and no-polycose saccharin gelatins and then with no-polycose fluids. The polycose and saccharin gelatin tests were balanced for order, and the fluid test was conducted last.

**Figure 7:**
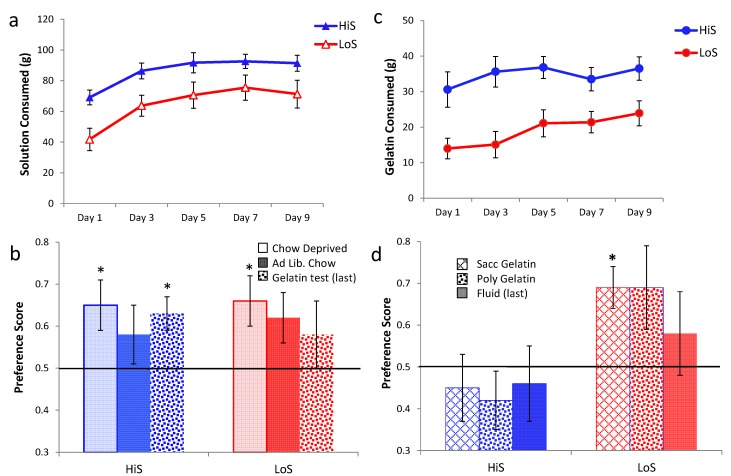
Intake during conditioning (polycose/ethanol solution in Experiment 7a and saccharin/ethanol gelatin in Experiment 7b, respectively, Panels **a** and **c**). Ethanol-paired flavor preference is shown in Panels **b** and **d**.

Intake of ethanol/polycose solution during conditioning in Experiment 7a is shown in Figure 7a. HiS rats drank more than did LoS rats, and intake increased over days. A line × day ANOVA yielded main effects of day and line, *F*(1,22) = 5.75, and day, *F*(4,88) = 38.52.

Ethanol-paired flavor preference is shown in Figure 7b. When chow deprived, both HiS and LoS rats expressed a significant preference for the ethanol-paired flavor in solution, *t*s(11) 2.47 and 2.40, respectively. The preference also was significant among HiS rats in the gelatin test, *t*(11) = 3.45. During *ad lib*. testing, preference scores did not differ significantly from 0.5; that preference therefore was less robust than observed previously for both HiS and LoS rats tested during *ad lib.* feeding [19,21], perhaps indicating some reduction due to use of the polycose vehicle during conditioning or to variability arising from the order-balanced re-testing. At any rate, both lines leaned toward preference, and not aversion, in all three tests when conditioned with polycose solutions. Also of note, HiS rats expressed an ethanol-paired flavor preference in the gelatin test, demonstrating that expression of an ethanol-flavor association is not always limited to the conditioning medium.

Intake of no-polycose saccharin/ethanol gelatin during conditioning in Experiment 7b is shown in Figure 7c. One LoS rat, who ate an average amount of ethanol gelatin but rejected most of the no-ethanol gelatin ration, was excluded from the following analyses. HiS rats consumed more saccharin gelatin than did LoS rats. Although intake by the latter trends upwards over days, no effects in a line × day ANOVA were significant other than the line main effect, *F*(1,21) = 17.84. Ethanol-paired flavor preference is shown in Figure 7d. LoS rats preferred the ethanol-paired flavor in gelatin but not fluid; within-group variability was lower for saccharin than for polycose gelatin. HiS rats were indifferent regardless of test medium. A line × test medium ANOVA yielded a main effect of line, *F*(1,21) = 4.33. Comparison of each mean to 0.5 confirmed LoS rats’ preference for the ethanol-paired flavor in saccharin gelatin, *t*(9) = 3.78. (Note: The excluded LoS rat’s preference for the ethanol-paired flavor was 0.95, so including her would only have increased deviation of the mean from 0.5). Their preference was not significant in polycose gelatin or fluid or in any of the three tests among HiS rats.

The yoking direction in Experiment 7b (unlimited ethanol saccharin gelatin, matched no-ethanol saccharin gelatin rations) was an educated guess based on the preceding experiments and a small pilot study that showed both were acceptable in single-jar tests. However, more rats left a higher proportion of their no-ethanol rations uneaten than we had seen in prior experiments. Followup analyses were conducted to determine whether ration-leaving—*i.e.*, relatively less exposure to the no-ethanol flavor—might have differentially biased LoS rats’ preference toward the ethanol-paired flavor. First, a line × day ANOVA was conducted on leftover ration amounts. It yielded no significant effects. Then, intercorrelations between amount of leftover gelatin and flavor preference in the three tests were assessed separately in each line with Pearson’s *r*. Among LoS rats, ethanol-paired flavor preference in the saccharin gelatin and fluid tests were strongly positively correlated, *r*(8) = 0.88, but neither was correlated with preference in the polycose gelatin test; none of the three preference scores was correlated with amount of leftover gelatin on any ration trial (*P*s > 0.13–0.93). Results were different for HiS rats: Preference in the polycose gelatin test was strongly correlated with preference in both the saccharin gelatin and the fluid test, *r*s(11) = 0.84 and 0.74, respectively, and the correlation between saccharin gelatin and fluid preferences was nearly significant, *r*(11) = 0.54, *P* = 0.055. Moreover, amount of leftover gelatin on the last ration day (and not before) predicted ethanol-paired flavor preference in both the saccharin and polycose gelatin tests, *r*s(11) = 0.56 and 0.63, respectively, but not in the fluid test. 

The greater stability of individual HiS rats’ relative flavor intake across contexts (*i.e.*, successive and simultaneous flavor presentations, three test media) suggests a difference in the basis on which HiS and LoS rats are responding to the two flavors, and not just a difference in the overall strength of associative conditioning or its expression. Lower reactivity to the relative novelty of test media among HiS rats is one possibility [21]. Alternatively, the greater stability in the HiS rats’ preference scores might reflect conditioning based on ethanol or stronger unconditioned flavor preferences; it is impossible to tell from these data because ethanol pairing and flavor (cherry, grape) are perfectly confounded at the individual level. That ration leftovers achieved significance as a predictor of HiS rats’ preference only at the end of conditioning hints at differential conditioning. Experiment 10 (below) explored the last idea.

## 4. Experiments 8–10: Unconditioned Flavor Test Series

Many results in the foregoing gelatin experiments were surprising in light of our prior results using solutions, prompting us to examine rats’ intake of unflavored (*i.e.*, no Koolaid added) polycose/ethanol mixtures, in gelatin and in solution. Whereas 10% polycose and 4% ethanol are both preferred to water by our rats [16,37] as well as others’ [7,38], generally modest intakes of conditioning gelatins and the need to reverse the yoking procedure in Experiment 5 suggested that 4% ethanol functions as an adulterant when added to a polycose medium. The following experiments explored whether this is the case and, if so, whether it holds equally for HiS and LoS rats.

### 4.1. Experiment 8

**Figure 8:**
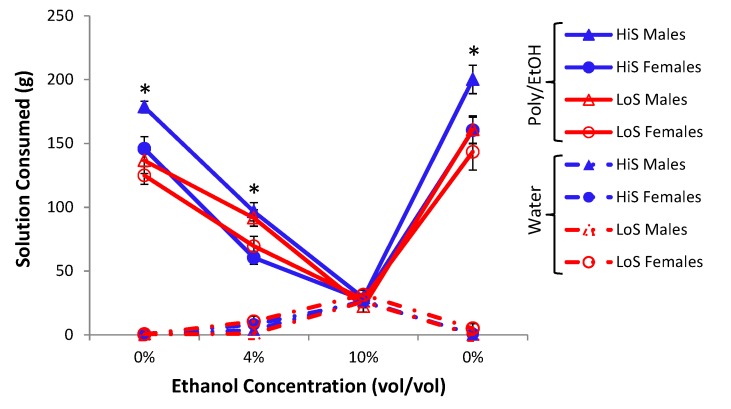
Intake of water and 10% polycose/ethanol solution as a function of ethanol concentration. Asterisks (*) indicate the ethanol concentrations at which all four groups drank more polycose/ethanol solution than water.

HiS and LoS female and male rats (*n*s = 13 or 14) were given 10% polycose solution in four successive 24-h two-bottle tests (*versus* water). Ethanol was added to the solution in the following order: 0%, 4%, 10%, 0%. Solution intake is shown in Figure 8. Overall, HiS rats drank more polycose solution than did LoS rats, but both lines drank less polycose solution when ethanol was added to it. At 10% ethanol, both lines drank no more polycose solution than they did water. A line × sex × solution (polycose, water) × test day ANOVA yielded a main effect of line, *F*(1,49) = 6.80, and many other significant effects and interactions.

For simplicity sake, we interpret here only the highest order interaction involving line: line × solution × test day, *F*(3,147) = 5.38. Bonferroni-adjusted contrasts comparing polycose to water intake by each line on each test day confirmed that both lines drank significantly more polycose solution than water on all test days except the 10% ethanol test day. Significant effects involving sex did not involve line and were ordinal (*i.e.*, the pattern was identical for males and females in both lines, with larger changes in intake among the larger males). Followup one-sample *t* tests comparing each group’s mean polycose solution preference score to 0.5 yielded a significant preference for all four groups on every test except 10% ethanol. High intake by all groups on the final 0% ethanol test shows that suppression of polycose intake by ethanol was not an artifact of prior test days.

Although 4% ethanol by itself is preferred to water, it reduces intake of 10% polycose solution by at least half. At 10% ethanol, intake of ethanol *per se* is about the same as at 4% ethanol, but preference for the polycose/ethanol solution over water is eliminated. Polycose’s palatable, starchy taste [39] makes it a “palatable vehicle” for ethanol in work with rats [31,32]—for instance, whereas our and other rats reject 10% ethanol in favor of water, here they did not reject 10% ethanol in a polycose vehicle in favor of water. However, the reverse does not hold: Ethanol is not a palatable vehicle for polycose, even at a low ethanol concentration that is by itself acceptable and palatable.

### 4.2. Experiment 9

**Figure 9:**
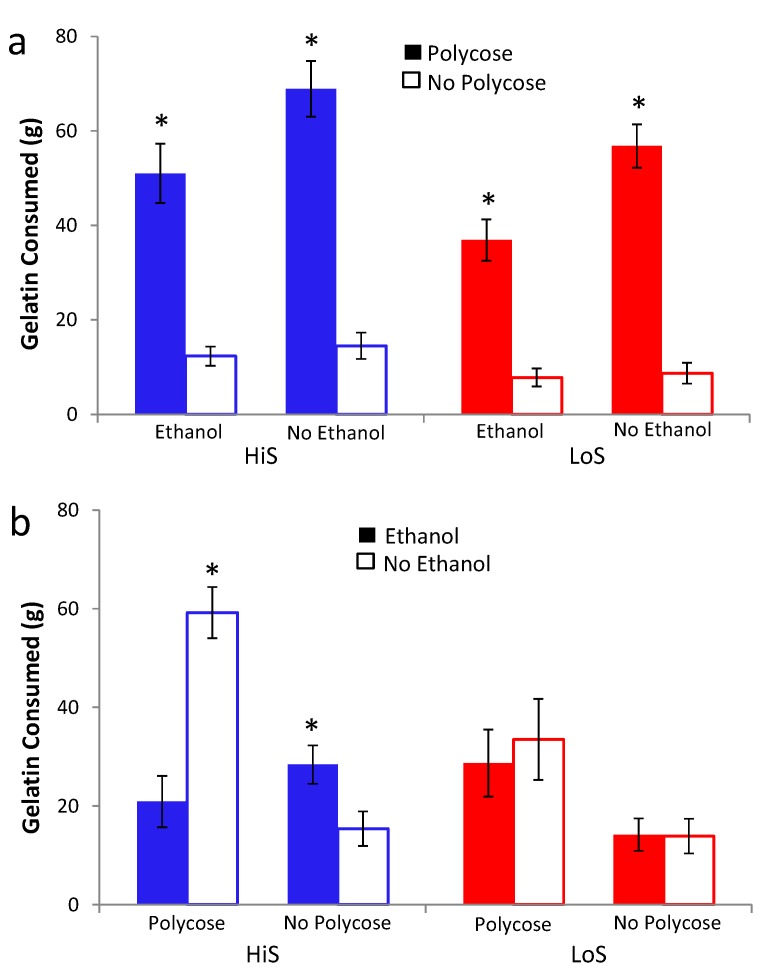
Gelatin intake in a two-jar choice between (**a**) 10% polycose and no-polycose gelatins (Experiment 9a) when both contain 4% ethanol or when neither contains ethanol, and (**b**) 4% ethanol or no-ethanol gelatins (Experiment 9b) when both contain 10% polycose or when neither contains polycose. Intake differing significantly from the other simultaneously available gelatin is marked with an asterisk (*).

Responses to polycose/ethanol mixtures in gelatin were examined in Experiment 9. Experiment 9a examined polycose preference as a function of whether the gelatin contained ethanol, and Experiment 9b examined ethanol preference as a function of whether the gelatin contained polycose. In Experiment 9a, male HiS and LoS rats (*n*s = 14) were given a choice 0% and 10% polycose gelatin. They were tested twice, once with both gelatins containing 4% ethanol and once with neither containing ethanol, with order balanced. The caloric density of gelatin without ethanol or polycose was 0.11 kcal/g. As shown in Figure 9a, gelatin intake was higher overall among HiS rats, but the lines otherwise were similar: Both ate more total gelatin during the test on which gelatins did not contain ethanol, and both strongly preferred polycose to no-polycose gelatin regardless of whether the gelatins contained ethanol. A line × polycose/no-polycose × ethanol/no-ethanol medium ANOVA yielded main effects of line, *F*(1,26) = 10.36, polycose/no-polycose, *F*(1,26) = 10.95, and medium, *F*(1,26) = 266.11, and an ordinal polycose/no-polycose × medium interaction, *F*(1,26) = 7.94. Similarly to response to polycose/ethanol solutions in Experiment 8, ethanol reduced polycose gelatin intake in both lines. However, polycose gelatin still was preferred to no-polycose gelatin even when both contained 4% ethanol. 

In Experiment 9b, male HiS and LoS rats (*n*s = 14) were given a choice between gelatin containing 0% or 4% ethanol. They were tested twice, once with both gelatins containing 10% polycose and once with no polycose, with order balanced. As shown in Figure 9b, HiS rats preferred polycose gelatin “straight”: When both gelatins contained polycose, they ate nearly three times more no-ethanol gelatin than ethanol gelatin. When neither gelatin contained polycose, they ate more 4% ethanol than no-ethanol gelatin. LoS rats ate more when both gelatins contained polycose but, interestingly, ate equal amount of ethanol and no-ethanol gelatin regardless of whether the gelatins contained polycose. 

A line × ethanol/no-ethanol × polycose/no-polycose medium ANOVA yielded line and medium main effects and a three-way interaction, *F*s(1,26) = 10.53, 143.95, and 5.86, respectively. Bonferroni-adjusted contrasts comparing ethanol to no-ethanol gelatin intake for each line with each medium confirmed that for HiS, EtOH < no EtOH in a polycose medium whereas EtOH > no EtOH in a no-polycose medium. No other differences were significant. Calculation of preference scores and comparisons of those means to 0.5 yielded identical conclusions.

HiS rats’ preference for 4% *versus* 0% ethanol in gelatin concurs with what we have previously observed for both lines using 4% ethanol solution *versus* water [16]. LoS rats’ indifference to 4% *versus* 0% ethanol gelatin does not. Apparently, something about the flavor or mouthfeel of gelatin, how gelatin affects ethanol absorption or distribution, or the act of eating rather than drinking eliminates LoS rats’—but not HiS rats’—tendency to choose 4% over 0% ethanol.

### 4.3. Experiment 10

**Figure 10:**
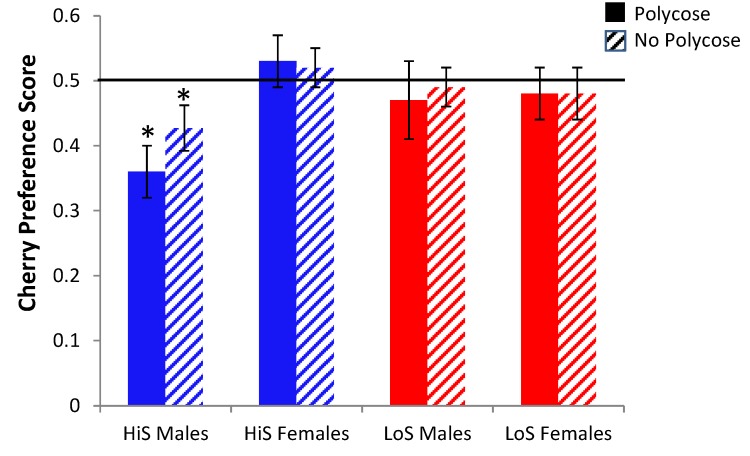
Unconditioned preference for cherry over grape flavored gelatin, tested once with 10% polycose and once without polycose, in Experiment 10. Mean preference scores differing significantly from 0.5 are indicated with an asterisk (*).

Using aqueous solutions in previous research, we observed no differences between rats receiving cherry-alcohol pairing and those receiving grape-alcohol pairing. In light of discrepancies between those studies and the present results with gelatin, we thought it wise to find out whether one or both lines prefer cherry and grape equally in gelatin. HiS and LoS females and males (*n*s = 21 or 22) were given a choice between cherry and grape flavored gelatin, once with both containing 10% polycose and once with neither containing polycose, with order balanced. Cherry preference is shown in Figure 10. HiS males preferred grape to cherry regardless of whether the gelatin contained polycose. 

A line × sex × polycose/no-polycose ANOVA yielded no significant effects. Comparison of each mean to 0.5 yielded significant results only for HiS males, *t*s(21) = 3.22 and 2.07, respectively.

Why HiS males prefer grape to cherry flavored gelatin is unclear. Both KoolAid flavors contain citric acid, to which HiS and LoS rats are equally averse [37]. These samples were unusually large with many litters represented so these results are unlikely to be a fluke. Luckily, in this particular within-rat procedure with ethanol-paired flavor assignments balanced, their choosiness does not strongly bias the results: Although having cherry paired with ethanol might tend to reduce preference for the (unpreferred) ethanol-paired flavor in HiS males, a comparable number of HiS males had grape paired with ethanol, which should tend to enhance preference for the (preferred) ethanol-paired flavor. If anything, the unconditioned flavor preference might be expected to increase variability among HiS males, but that is not apparent in these results. Controlling for strain differences in unconditioned flavor preferences, however, would be important in procedures involving between-group flavor assignments.

## 5. Discussion

Two key conclusions emerge from this work. First, studying flavor conditioning using gelatin as a medium requires careful consideration of technical issues that, in our experience, are less likely to arise when solutions are used. In several instances, our guesses about the relative acceptability of and preferences for substances presented in gelatin, educated by prior work with solutions, were wrong. Examples include HiS male rats’ slight but significant preference for grape over cherry in gelatin, LoS male rats’ indifference to 4% ethanol in gelatin (with or without polycose), and surprising variations in unconsumed gelatin rations. A practical consequence was that our decisions about the direction of yoking (*i.e.*, of ethanol or no-ethanol gelatin, which would be available *ad lib*. and which would be rationed) did not always yield the degree of flavor exposure matching that is more easily achieved with solutions. Investigators wishing to study learning with gelatin as a medium will do well to conduct substantial pilot testing to set parameters rather than generalizing from one procedure or rat strain to another.

Second, when it comes to flavor conditioning, ethanol vehicle matters. Gelatin is an effective medium for increasing rats’ voluntary ethanol intake, but it is not an effective medium with which to condition an ethanol-paired flavor preference. Parameters used in one or more of these gelatin conditioning experiments should have favored expression of a reliable preference for a flavor paired with ethanol, as we and others have observed using solutions: Ethanol was delivered as a modest total dose consumed over hours rather than as bolus injection or gavage [9], ethanol concentrations varied in palatability [40], ethanol gelatin offered a caloric advantage over no-ethanol gelatin [8,35], and some rats were conditioned and/or tested hungry [8,10,11,12]. Yet the result across all parametric variations was indifference or aversion to the flavor paired with ethanol in gelatin for HiS rats. LoS rats were somewhat less prone to aversion: When reasonable yoking was achieved, LoS rats twice were indifferent when HiS counterparts were averse (Experiments 2 and 6) and twice showed a preference when HiS counterparts were indifferent (Experiments 5a and 7b). Although one of the LoS rats’ preferences failed to replicate and the other awaits replication, that HiS rats conditioned using gelatin never expressed a significant preference is noteworthy, especially given that they express ethanol-paired flavor preferences conditioned with fluid more robustly across procedural variations than do LoS rats [21], including generalization to flavored gelatins (Experiment 7a). Eating ethanol in gelatin and drinking it in solution do not appear to be not equivalent learning experiences.

The elusiveness of ethanol-paired flavor preference using a gelatin vehicle remains to be explained. Although unconditioned flavor hedonics or caloric advantage appear to be promising candidates, neither provides a satisfying account without consideration of vehicle. True, ethanol functions as an “adulterant” in polycose gelatin, reducing its consumption even at a concentration (4%) that is preferred or treated with indifference in no-polycose gelatin (Experiment 9b). And rats expressed indifference to or preference for the flavor associated with 4% ethanol/polycose gelatin but aversion to the flavor associated with 10% ethanol/polycose gelatin, even though the doses of ethanol consumed were roughly the same (Experiment 5). These results might seem to implicate the unconditionally negative hedonic value of the ethanol/polycose compound in choices expressed later. However, rats do learn and express conditioned preferences for unconditionally aversive flavors [40,41,42]. Moreover, ethanol also reduces drinking of polycose *solution* (Experiment 8), yet we replicated ethanol-paired flavor preference in both HiS and LoS rats using polycose solution as an ethanol vehicle (Experiment 7a). The latter finding also is contrary to the idea that the caloric advantage of polycose/ethanol gelatin over polycose/no-ethanol gelatin *per se* was insufficient to support preference learning, and might even account for aversion [10,11,43]. The limited importance of to-be-conditioned flavors and the effectiveness of flavor-nutrient conditioning with polycose solution, despite the dramatic increase in its caloric value compared to aqueous solution, also make it unlikely that gelatin’s taste, protein, or modest calories completely scuttle preference conditioning, especially among HiS rats.

The present experiments were not designed to study generalization. Testing always started with the same medium (gelatin or solution) as had been used in conditioning, with a final test with the alternate medium added on. With that caveat, these results hint at asymmetry in gelatin/solution generalization. Mehiel [13] argued that nutrient-paired flavor preferences reflect a shift in hedonic value that generalizes broadly across flavor media and environmental contexts. However, only evidence concerning solution (conditioning)→chow mash (test) generalization was cited, and generalization in that direction could be more robust than the converse. In none of the seven gelatin-conditioning experiments reported here was a statistically significant flavor aversion or preference expressed in the fluid test, regardless of whether that test was after one or two gelatin tests (extinction trials). On the other hand, in the single experiment with solution conditioning, the HiS rats’ preference for a flavor paired with ethanol in polycose solution did generalize to gelatin, despite that test occurring after two gelatin (extinction) tests. Further studies with balancing of test order are needed to determine whether ethanol-paired flavor preferences and aversions conditioned with gelatin generalize less broadly than the converse, at least in some rat strains, as these results might suggest.

The striking contrast in behavior toward a flavor paired with ethanol in gelatin *versus* solution ultimately might be best understood from a behavior-systems perspective. To be sure, no bright line separates eating from drinking, especially in omnivores such as rats and humans: Foods can be drunk (blood, milk, other liquid diets) and water can be eaten (in prey, fruit, ice), and diverse consummatory behaviors share some associative learning and motivational mechanisms [44,45,46,47]. Nonetheless, eating and drinking are different behaviors that have been shaped by selective pressures related predominantly to, respectively, nutrients and hydration, and they have dissociable motoric, motivational, and neural mechanisms [48,49,50]. For instance, mammals’ need for water is less elastic than is the need for calories and, given the greater range of edibles in most environments relative to liquids, pressure to choose well among foods likely has been greater than choosing among fluids, especially for omnivores [51]. Such differences might be expected to yield asymmetries in learning based on drinking *versus* eating. Gelatin is a useful tool for exploring such asymmetries because its highly soluble nature minimizes differences from solutions in terms of mechanical transformation of the food by the gut, making behavioral differences (e.g., use of teeth, food handling) relatively more important.

Ontogenetically, asymmetry could derive from the fact that all mammals’ first food is a liquid nutrient—milk (or milk substitute). Young mammals begin learning about foods in their environment via flavors in mothers’ milk [52]. Beginning life by drinking milk may prepare mammals to generalize from fluids to solid foods later, more so than the reverse. Ethanol is interesting in developmental and ethological contexts because, like milk, it is also is a liquid nutrient, and as such is both food and beverage. Interestingly, like milk, ethanol has opioid-mediated appetitive properties in rat pups neonatally; its aversive properties emerge by 12 days of age [53]. Milk also is an effective vehicle for boosting ethanol intake by human adolescents, even when alcohol’s taste is not effectively masked [27].

## 6. Conclusions

Our difficulty in obtaining reliable preferences for a flavor paired with ethanol in gelatin, especially among HiS rats, might be rooted in the evolution and development of systems specialized for meeting nutritional *versus* hydrational challenges. One implication is that conclusions obtained with flavored solutions or foods should be generalized to the other medium cautiously. In particular, eating ethanol might influence dietary choices in a more flavor- and medium-specific way than drinking it does. Another implication is that individual differences in sensitivity to nutrients and metabolic status can manifest differently in eating *versus* drinking paradigms. The neural mechanisms through which eating *versus* drinking ethanol can yield different effects warrant further consideration.

A final implication concerns “jello shot” ingestion outside of laboratories. Clearly, it boosts ethanol intake on college campuses. It might not, however, readily condition generalizable preferences for flavors associated with ethanol. Therefore, while consuming ethanol-spiked gelatin might tend to increase consumption of ethanol-spiked gelatin, it might not increase proclivity to consume ethanol as effectively as does drinking beer, wine, or cocktails. 
